# 7-Hy­droxy­indan-1-one

**DOI:** 10.1107/S1600536811009718

**Published:** 2011-03-19

**Authors:** Kew-Yu Chen, Yuh-Sheng Wen, Tzu-Chien Fang, Yuan-Jay Chang, Ming-Jen Chang

**Affiliations:** a Department of Chemical Engineering, Feng Chia University, 40724 Taichung, Taiwan; b Institute of Chemistry, Academia Sinica, 115 Taipei, Taiwan

## Abstract

In the title compound, C_9_H_8_O_2_, an intra­molecular O—H⋯O hydrogen bond generates an *S*(6) ring. The dihedral angle between the mean plane of the *S*(6) ring and the benzene ring is 1.89 (2)°. In the crystal, inversion-related mol­ecules are linked by pairs of O—H⋯O hydrogen bonds, forming a cyclic dimers with *R*_2_^2^(12) graph-set motif. Weak inter­molecular C—H⋯O_carbon­yl_ and C—H⋯O_hy­droxy_ hydrogen bonds link the dimers into chains along [010], generating two *C*(6) motifs that overlap three C atoms, forming *R*_2_^2^(8) ring motifs.

## Related literature

For the spectroscopy and the dynamic processes related to the intramolecular proton transfer of the title compound, see: Aquino *et al.* (2005 ▶); Chou *et al.* (1991 ▶); Nagaoka *et al.* (1984 ▶); Nishiya *et al.* (1986 ▶). For its preparation, see: Tadić *et al.* (1988 ▶). For related structures, see: Li *et al.* (2007 ▶); Saeed *et al.* (2007 ▶). For graph-set theory, see: Bernstein *et al.* (1995 ▶).
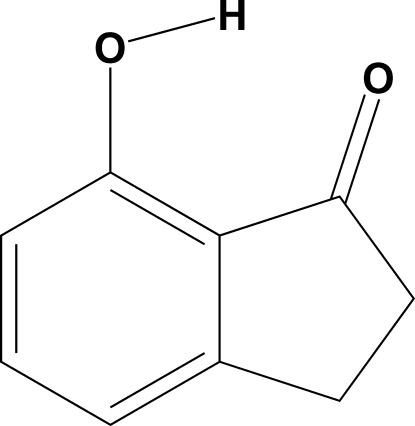


## Experimental

### 

#### Crystal data

C_9_H_8_O_2_
*M* *_r_* = 148.15
Monoclinic, 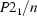

*a* = 7.3457 (3) Å
*b* = 13.3767 (5) Å
*c* = 7.3693 (3) Å
β = 108.584 (2)°
*V* = 686.36 (5) Å^3^
*Z* = 4
Mo *K*α radiation
μ = 0.10 mm^−1^
*T* = 100 K
0.28 × 0.24 × 0.24 mm

#### Data collection

Bruker SMART CCD area-detector diffractometer
Absorption correction: multi-scan (*SADABS*; Bruker, 2001 ▶) *T*_min_ = 0.676, *T*_max_ = 0.745
5437 measured reflections
1400 independent reflections
1252 reflections with *I* > 2σ(*I*)
*R*_int_ = 0.022

#### Refinement

*R*[*F* ^2^ > 2σ(*F* ^2^)] = 0.033
*wR*(*F* ^2^) = 0.089
*S* = 1.04
1400 reflections
133 parameters
All H-atom parameters refined
Δρ_max_ = 0.28 e Å^−3^
Δρ_min_ = −0.18 e Å^−3^

### 

Data collection: *SMART* (Bruker, 2001 ▶); cell refinement: *SAINT* (Bruker, 2001 ▶); data reduction: *SAINT*; program(s) used to solve structure: *SHELXS97* (Sheldrick, 2008 ▶); program(s) used to refine structure: *SHELXL97* (Sheldrick, 2008 ▶); molecular graphics: *ORTEP-3 for Windows* (Farrugia, 1997 ▶); software used to prepare material for publication: *WinGX* (Farrugia, 1999 ▶).

## Supplementary Material

Crystal structure: contains datablocks I, global. DOI: 10.1107/S1600536811009718/si2340sup1.cif

Structure factors: contains datablocks I. DOI: 10.1107/S1600536811009718/si2340Isup2.hkl

Additional supplementary materials:  crystallographic information; 3D view; checkCIF report

## Figures and Tables

**Table 1:** Hydrogen-bond geometry (Å, °)

*D*—H⋯*A*	*D*—H	H⋯*A*	*D*⋯*A*	*D*—H⋯*A*
O2—H2⋯O1	0.880 (17)	2.182 (18)	2.899 (1)	138 (1)
O2—H2⋯O1^i^	0.880 (17)	2.219 (14)	2.864 (1)	130 (1)
C1—H1*B*⋯O2^ii^	0.985 (14)	2.519 (14)	3.478 (1)	164 (1)
C4—H4⋯O1^iii^	0.964 (16)	2.527 (16)	3.467 (1)	165 (1)

Symmetry codes: (i) 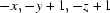; (ii) 
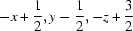
; (iii) 
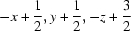
.

**Fig. 1.**
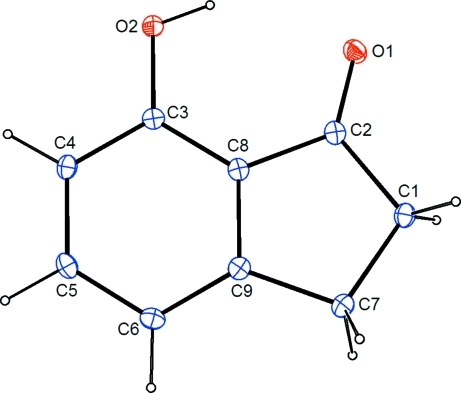
The molecular structure of the title compound, showing 50% probability displacement ellipsoids.

**Fig. 2.**
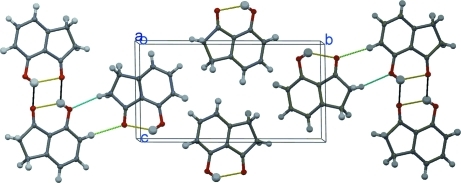
A section of the crystal packing of the title compound, viewed down the a axis.

## supplementary crystallographic information

### Comment

The excited-state intramolecular proton transfer (ESIPT) reaction of 7-hydroxy-1-indanone (7HIN) has been investigated for past decades (Aquino *et al.*, 2005; Nagaoka *et al.*, 1984; Nishiya *et al.*, 1986), which incorporates transfer of a hydroxy proton to the carbonyl oxygen through a intramolecular six-membered-ring hydrogen-bonding system (Chou *et al.*, 1991).

The *ORTEP* diagram of the title compound is shown in Figure 1. The indane moiety is essentially planar (r.m.s. deviation for the nine C atoms = 0.014 Å), which is consistent with previous studies (Li, *et al.*, 2007; Saeed *et al.*, 2007). 7HIN possesses a intramolecular six-membered ring hydrogen bond, which generates an *S*(6) ring motif. The dihedral angle between the mean plane of the *S*(6) ring and the mean plane of the benzene ring is 1.89 (2)°. This, together with 2.182 (18) Å of O2—H2···O1 distance and 138 (1)° of O2—H2—O1 (Table 1), strongly supports the *S*(6) ring formation. (Bernstein *et al.*, 1995). In the crystal structure, two inversion related molecules are linked by dual O—H···O hydrogen bonds (black dashed line) to form a cyclic dimer of *R*_2_^2^(12) ring system (Fig. 2). Furthermore, weak intermolecular C4—H4···O1 (green dashed line) and C1—H1B···O2 (blue dashed line) hydrogen bonds link the dimers into the chains along [0 1 0], generating two C(6) motifs that overlap three C atoms (C2, C8 and C3) to form *R*_2_^2^(8) ring motifs.

### Experimental

7-hydroxy-1-indanone was purchased from Sigma-Aldrich (>97% purity) and used as received without further purification. White needle-shaped crystals suitable for the crystallographic studies reported here were isolated over a period of two weeks by slow evaporation from a cyclohexane solution.

### Refinement

H atoms bonded to O and C atoms were located in a difference electron density map and refined freely with respective distances of 0.88 (2) Å for O—H, and for C—H in the range 0.95 (2) - 1.01 (1) Å. The freely refined *U*_iso_(H) were found in ranges between 0.019 (3) and 0.024 (4) Å^-2^ (bound to C atoms), for the hydroxy H atom a value of 0.044 (5) Å^-2^ was observed.

### Crystal data

**Table:**

C_9_H_8_O_2_	*F*(000) = 312
*M**_r_* = 148.15	*D*_x_ = 1.434 Mg m^−^^3^
Monoclinic, *P*2_1_/*n*	Mo *K*α radiation, λ = 0.71073 Å
Hall symbol: -P 2yn	Cell parameters from 2865 reflections
*a* = 7.3457 (3) Å	θ = 3.3–26.4°
*b* = 13.3767 (5) Å	µ = 0.10 mm^−^^1^
*c* = 7.3693 (3) Å	*T* = 100 K
β = 108.584 (2)°	Prism, colourless
*V* = 686.36 (5) Å^3^	0.28 × 0.24 × 0.24 mm
*Z* = 4	

### Data collection

**Table:**

Bruker SMART CCD area-detector diffractometer	1400 independent reflections
Radiation source: fine-focus sealed tube	1252 reflections with *I* > 2σ(*I*)
graphite	*R*_int_ = 0.022
φ and ω scans	θ_max_ = 26.4°, θ_min_ = 3.1°
Absorption correction: multi-scan (*SADABS*; Bruker, 2001)	*h* = −9→9
*T*_min_ = 0.676, *T*_max_ = 0.745	*k* = −16→16
5437 measured reflections	*l* = −9→7

### Refinement

**Table:**

Refinement on *F*^2^	Secondary atom site location: difference Fourier map
Least-squares matrix: full	Hydrogen site location: inferred from neighbouring sites
*R*[*F*^2^ > 2σ(*F*^2^)] = 0.033	All H-atom parameters refined
*wR*(*F*^2^) = 0.089	*w* = 1/[σ^2^(*F*_o_^2^) + (0.0465*P*)^2^ + 0.2373*P*] where *P* = (*F*_o_^2^ + 2*F*_c_^2^)/3
*S* = 1.04	(Δ/σ)_max_ < 0.001
1400 reflections	Δρ_max_ = 0.28 e Å^−^^3^
133 parameters	Δρ_min_ = −0.18 e Å^−^^3^
0 restraints	Extinction correction: *SHELXL97* (Sheldrick, 2008), Fc^*^=kFc[1+0.001xFc^2^λ^3^/sin(2θ)]^-1/4^
Primary atom site location: structure-invariant direct methods	Extinction coefficient: 0.014 (4)

### Special details

**Table:**

Geometry. All e.s.d.'s (except the e.s.d. in the dihedral angle between two l.s. planes) are estimated using the full covariance matrix. The cell e.s.d.'s are taken into account individually in the estimation of e.s.d.'s in distances, angles and torsion angles; correlations between e.s.d.'s in cell parameters are only used when they are defined by crystal symmetry. An approximate (isotropic) treatment of cell e.s.d.'s is used for estimating e.s.d.'s involving l.s. planes.
Refinement. Refinement of *F*^2^ against ALL reflections. The weighted *R*-factor *wR* and goodness of fit *S* are based on *F*^2^, conventional *R*-factors *R* are based on *F*, with *F* set to zero for negative *F*^2^. The threshold expression of *F*^2^ > σ(*F*^2^) is used only for calculating *R*-factors(gt) *etc*. and is not relevant to the choice of reflections for refinement. *R*-factors based on *F*^2^ are statistically about twice as large as those based on *F*, and *R*- factors based on ALL data will be even larger.

### Fractional atomic coordinates and isotropic or equivalent isotropic displacement parameters (Å^2^)

**Table:**

	*x*	*y*	*z*	*U*_iso_*/*U*_eq_	
O1	0.08166 (12)	0.42309 (6)	0.66091 (12)	0.0198 (2)	
O2	0.23230 (12)	0.62427 (6)	0.67482 (12)	0.0187 (2)	
C1	0.13603 (16)	0.35050 (9)	0.97988 (17)	0.0164 (3)	
C2	0.14647 (15)	0.42957 (8)	0.83566 (17)	0.0147 (3)	
C3	0.28549 (15)	0.60687 (8)	0.86582 (17)	0.0141 (3)	
C4	0.38227 (16)	0.68108 (9)	0.99239 (17)	0.0158 (3)	
C5	0.43377 (16)	0.66351 (9)	1.18845 (17)	0.0168 (3)	
C6	0.39144 (16)	0.57428 (9)	1.26467 (17)	0.0168 (3)	
C7	0.23864 (17)	0.39569 (9)	1.17926 (18)	0.0170 (3)	
C8	0.24541 (15)	0.51652 (8)	0.94170 (16)	0.0139 (3)	
C9	0.29761 (15)	0.49992 (8)	1.13835 (17)	0.0143 (3)	
H1A	0.001 (2)	0.3364 (10)	0.9591 (19)	0.019 (3)*	
H1B	0.1971 (19)	0.2884 (10)	0.9566 (19)	0.019 (3)*	
H2	0.164 (2)	0.5745 (14)	0.609 (2)	0.044 (5)*	
H4	0.414 (2)	0.7437 (12)	0.945 (2)	0.026 (4)*	
H5	0.5008 (19)	0.7159 (10)	1.2762 (19)	0.019 (3)*	
H6	0.425 (2)	0.5649 (11)	1.398 (2)	0.024 (4)*	
H7A	0.154 (2)	0.3975 (11)	1.264 (2)	0.024 (4)*	
H7B	0.354 (2)	0.3563 (10)	1.250 (2)	0.021 (4)*	

### Atomic displacement parameters (Å^2^)

**Table:**

	*U*^11^	*U*^22^	*U*^33^	*U*^12^	*U*^13^	*U*^23^
O1	0.0216 (5)	0.0187 (5)	0.0167 (5)	−0.0005 (3)	0.0025 (4)	−0.0018 (3)
O2	0.0228 (5)	0.0171 (4)	0.0146 (5)	−0.0036 (3)	0.0038 (3)	0.0006 (3)
C1	0.0153 (6)	0.0142 (6)	0.0200 (6)	0.0005 (4)	0.0061 (5)	0.0003 (5)
C2	0.0113 (5)	0.0144 (6)	0.0187 (6)	0.0021 (4)	0.0051 (4)	−0.0008 (4)
C3	0.0117 (5)	0.0157 (6)	0.0151 (6)	0.0024 (4)	0.0043 (4)	0.0009 (4)
C4	0.0140 (5)	0.0130 (5)	0.0204 (7)	0.0002 (4)	0.0057 (5)	0.0005 (4)
C5	0.0143 (5)	0.0153 (6)	0.0195 (6)	0.0006 (4)	0.0035 (5)	−0.0044 (5)
C6	0.0159 (6)	0.0198 (6)	0.0141 (6)	0.0024 (4)	0.0037 (5)	−0.0003 (5)
C7	0.0178 (6)	0.0154 (6)	0.0180 (6)	0.0008 (4)	0.0059 (5)	0.0019 (5)
C8	0.0110 (5)	0.0140 (5)	0.0170 (6)	0.0017 (4)	0.0050 (4)	−0.0010 (4)
C9	0.0118 (5)	0.0145 (6)	0.0173 (6)	0.0033 (4)	0.0054 (4)	0.0007 (4)

### Geometric parameters (Å, °)

**Table:**

O1—C2	1.2255 (14)	C4—C5	1.3919 (17)
O2—C3	1.3556 (14)	C4—H4	0.962 (16)
O2—H2	0.883 (19)	C5—C6	1.3958 (17)
C1—C2	1.5185 (16)	C5—H5	0.974 (14)
C1—C7	1.5448 (17)	C6—C9	1.3864 (16)
C1—H1A	0.976 (15)	C6—H6	0.945 (15)
C1—H1B	0.984 (14)	C7—C9	1.5184 (16)
C2—C8	1.4594 (15)	C7—H7A	1.011 (14)
C3—C4	1.3922 (16)	C7—H7B	0.993 (14)
C3—C8	1.4017 (16)	C8—C9	1.3937 (16)
			
C3—O2—H2	111.7 (11)	C4—C5—H5	118.7 (8)
C2—C1—C7	105.96 (9)	C6—C5—H5	118.6 (8)
C2—C1—H1A	107.5 (8)	C9—C6—C5	118.04 (11)
C7—C1—H1A	112.9 (8)	C9—C6—H6	121.0 (9)
C2—C1—H1B	109.8 (8)	C5—C6—H6	120.9 (9)
C7—C1—H1B	112.6 (8)	C9—C7—C1	104.72 (9)
H1A—C1—H1B	107.9 (11)	C9—C7—H7A	111.8 (8)
O1—C2—C8	125.41 (11)	C1—C7—H7A	112.6 (8)
O1—C2—C1	126.65 (10)	C9—C7—H7B	110.0 (8)
C8—C2—C1	107.94 (10)	C1—C7—H7B	111.6 (8)
O2—C3—C4	119.33 (10)	H7A—C7—H7B	106.3 (11)
O2—C3—C8	122.32 (10)	C9—C8—C3	121.94 (10)
C4—C3—C8	118.35 (11)	C9—C8—C2	110.78 (10)
C5—C4—C3	119.14 (11)	C3—C8—C2	127.28 (11)
C5—C4—H4	120.3 (9)	C6—C9—C8	119.80 (11)
C3—C4—H4	120.5 (9)	C6—C9—C7	129.62 (11)
C4—C5—C6	122.71 (11)	C8—C9—C7	110.57 (10)

### Hydrogen-bond geometry (Å, °)

**Table:**

*D*—H···*A*	*D*—H	H···*A*	*D*···*A*	*D*—H···*A*
O2—H2···O1	0.880 (17)	2.182 (18)	2.899 (1)	138 (1)
O2—H2···O1^i^	0.880 (17)	2.219 (14)	2.864 (1)	130 (1)
C1—H1B···O2^ii^	0.985 (14)	2.519 (14)	3.478 (1)	164 (1)
C4—H4···O1^iii^	0.964 (16)	2.527 (16)	3.467 (1)	165 (1)

Symmetry codes:  (i) −*x*, −*y*+1, −*z*+1; (ii) −*x*+1/2, *y*−1/2, −*z*+3/2; (iii) −*x*+1/2, *y*+1/2, −*z*+3/2.
